# Understanding Hearing Health: A Cross-Sectional Study of Determinants in a Metropolitan Area

**DOI:** 10.3390/healthcare11162253

**Published:** 2023-08-10

**Authors:** Francesca Pennino, Maddalena Di Lillo, Michele Sorrentino, Claudio Fiorilla, Antonio Parisi, Pasquale Domenico Mirizzi, Bruna De Simone, Paolo Montuori, Maria Triassi, Antonio Nardone

**Affiliations:** Department of Public Health, “Federico II” University, Via Sergio Pansini nº 5, 80131 Naples, Italy

**Keywords:** hearing health, KAP model, knowledge, attitude, behaviors

## Abstract

Hearing health prevention has emerged as a significant public health concern worldwide. With nearly two and a half billion people experiencing some degree of hearing loss, and around seven hundred million requiring medical intervention, the impact on global health is substantial. The economic burden is equally substantial, with estimated health costs reaching 980 billion dollars in the United States alone. To shed light on this issue, we conducted a survey-based cross-sectional study involving 1150 individuals. Utilizing multiple linear regression across three models, we aimed to explore the association between demographic variables and knowledge, attitude, and behaviors related to hearing health. In Model I, we observed a correlation between knowledge and several factors, including age, smoking habits, marital status, and education. In Model II, attitudes were found to associate with non-smoking habits, education, and knowledge. Model III revealed a statistically significant correlation between behaviors and age, gender, parenthood, knowledge, and attitudes. These findings emphasize the importance of targeted public health programs aimed at improving behaviors among the general population. Such interventions can be both effective and relatively inexpensive. By addressing these determinants, we can enhance overall hearing health in the community. Our study contributes valuable information about the knowledge, attitudes, and behaviors related to hearing health in the general population. Understanding these factors is crucial in developing evidence-based strategies to promote hearing health and prevent hearing loss effectively. As we continue to work towards better hearing health, the findings from this study can serve as a cornerstone for informed decision-making and successful intervention implementation.

## 1. Introduction

Affecting more than half a billion people, hearing loss is the most common sensory deficit globally [1,2]. Hearing loss has become a major concern for global health, impacting various aspects, such as communication and quality of life [3]. Indeed, hearing impairment leads to social isolation, loneliness, and stigma, influencing the number of years lived with a disability, and disability-adjusted life years [3,4,5].

The World Health Organization has projected that by 2050, almost 2.5 billion individuals will experience some level of hearing loss, with at least 700 million individuals requiring some form of hearing rehabilitation [3]. Furthermore, more than 1 billion young adults are at risk of permanent and preventable hearing loss due to unsafe hearing behaviors [6]. In the United States, approximately 22 million people are exposed to dangerous noise levels [7], and globally, harmful behaviors related to personal listening devices impact 0.67–1.35 billion adolescents and young adults [8].

In the European Region, roughly 190 million people have some level of hearing loss or deafness, which is equivalent to 20% of the population, meaning that one out of every five people in Europe has some form of hearing loss or deafness [3,9].

Hearing quality also affects health expenses, quantified at 980 billion US dollars globally [3]. This estimation helps to model the cost-effectiveness of interventions to prevent/address hearing loss and reinforce the case for investment [10].

There are numerous causes of hearing loss, including congenital, infectious, noise exposure through listening to music and in the workplace, age-related, traumatic, and immune-mediated causes [11,12]. However, it is important to note that age remains the most significant predictor of hearing loss among adults aged 20 to 69, with the highest prevalence occurring in the age group of 60 to 69 [13]. Additionally, it has been observed that men are almost twice as likely as women to experience hearing loss in this age group [13].

Although the causes of hearing loss are well-established, the existing literature indicates that adopting behaviors to safeguard hearing health is challenging for individuals [2,14]. As reported, 46.7% of American adults aged 18 years and older have reported some degree of hearing difficulty [15]. However, the prevalence of hearing loss in adults aged 20 to 69 declined over time. It was reported as 16% during the period1999–2004 [16], decreased to 14% during the period 2011–2012 [16]_,_ and further decreased to 7% during the period 2017–2020 [13].

To the best of our knowledge, there is a paucity of evidence regarding knowledge, attitude, and behaviors regarding hearing quality. Health behavior models were used to understand hearing conservation and its determinants, including attitudes and behaviors [17,18,19]. In 2014, Saunders analyzed hearing health in a sample of 235 participants between 18 and 80 years old but only focused on noise exposure damage and did not consider other causes of hearing loss [20]. Recently, Basheer et al. conducted a similar study but specifically focused on printing press workers, a high-risk population due to the exposition to high levels of noise that can damage hearing over time [21]. In conclusion, Almutairi, in 2022, conducted a study on knowledge and attitudes in Saudi Arabia, focusing on infant hearing loss [22].

Therefore, the purpose of the present study is to address the current lack of understanding about knowledge, attitudes, and behaviors related to hearing health in a large metropolitan area population. This study will provide valuable insights into how factors such as age, gender, and occupation influence hearing health knowledge, attitudes, and behaviors in different subgroups of the population. Those findings can be used to develop targeted public health interventions, improve hearing health awareness, and promote positive behaviors related to hearing conservation.

## 2. Materials and Methods

### 2.1. Setting and Sample 

This cross-sectional study administered questionnaires (Supplementary Materials) to adults residing in the metropolitan city of Naples (Italy), which has a population of 909,048 (ISTAT, 2022). The study was conducted from October 2022 to January 2023, using a snowball sampling method to select 1670 subjects from various settings, including universities, workplaces, and community centers. Out of those approached, 1155 participants willingly agreed to participate and returned fully completed questionnaires, resulting in a commendable response rate of 69.16%. To be eligible for inclusion, the participants had to be aged 18 and above and residing in the metropolitan area of Naples.

The sample size was determined using Slovin’s formula, aiming for a representative sample with a 3% margin of error and a 95% confidence interval. The calculation led to a final target of 1523 subjects. However, considering the possibility of non-response, the estimated total sample size was adjusted to 1066.

Table 1 presents the characteristics of the study population. The final sample comprised 1155 participants, resulting in an impressive response rate of 75.8%. Among the respondents, 53.3% were females and 46.7% were males. The mean age of the participants was 40.50 years, with a range of 18 to 86 years (S.D. ± 15.16). The largest age group was “under 30 years,” accounting for 39.4% of the participants, followed by “over 51 years,” comprising 28.8%. Regarding parenthood, 60.4% of the participants did not have children, while 39.6% were parents. Additionally, 59.5% of the respondents were in a relationship, and a majority of 64.2% did not smoke.

### 2.2. Procedures

During the study period, experienced interviewers submitted the questionnaire to the participants, as previously described [23,24,25]. 

The participants were thoroughly informed about the research’s typology, aims, and methodology, and the confidential handling of their collected data. Interviewers explicitly stated that they were representing the University of Naples “Federico II” while conducting the study. Importantly, the participants were assured of their right to withdraw from the study at any point without the need to provide a reason. Prior to proceeding with the interview, informed consent was obtained from all of the participants.

In conclusion, it was clarified that they could end their participation at any time without disclosing a reason. Consent was obtained prior to progressing with the interview. No founding was granted for participation. The present study was carried out in conformity with the Declaration of Helsinki.

### 2.3. Data Collection

The questionnaire was meticulously developed through collaborative meetings involving a diverse commission of physicians and other healthcare professionals. During this process, questions deemed irrelevant or unsuitable for the study’s objectives were carefully eliminated or replaced. To ensure the clarity and effectiveness of the questionnaire, a beta test was conducted with a separate group of 20 individuals, who were not included in the main study. This pretest allowed us to assess the participants’ comprehension of the questionnaire and make any necessary refinements.

*The first section of the questionnaire focused on gathering socio-demographic characteristics and other pertinent health-related information, such as gender, age, marital status, level of education, occupation, and smoking habits. In the second section, participants were probed about their knowledge, attitudes, and behaviors concerning hearing health. This section comprised a total of 32 questions. Responses for knowledge and attitudes were categorized into three options: “agree,” “uncertain,” and “disagree,” which were coded as 1, 2, and 3, respectively. On the other hand, behaviors were assessed with four response options: “yes/always”, “often”, “sometimes” and “never”, which were coded as 1, 2, 3 and 4, respectively.*

### 2.4. Statistical Analysis

The data obtained from the study were analyzed using the STATA MP v14.0 statistical software program (College Station, TX, USA). The analysis was performed in two stages. Firstly, descriptive statistics were used to summarize the basic information of the statistical units. Secondly, a multiple linear regression analysis (MLRA) was conducted across three models: Model I and Model II were partially adjusted, while Model III was fully adjusted.

To generate the knowledge, attitudes, and behaviors scores, individual scores from each section were summed up. The independent variables included in all of the models were: sex (1 = male, 2 = female), age (in years), education level (1 = primary school, 2 = middle school, 3 = high school, and 4 = university degree), marital status (1 = single; 2 = in a relationship), smoking habits (1 = smoker, 2 = non-smoker), and having children (1 = yes; 2 = no).

In Model II, the knowledge score was added to Model I, and in Model III, attitude was added to Model II. All statistical tests were two-tailed, and results were considered statistically significant if the *p*-values were less than or equal to 0.05.

## 3. Results

Table 2 presents the findings related to the respondents’ knowledge of hearing health. The results show that 68.8% of the participants correctly identified the ear as the organ responsible for hearing. Furthermore, 56.2% of the respondents did not agree that cotton buds are the safest way to clean ears. The study found that 67.4% of the respondents knew that otitis media is an ear disease, but less than half, 47%, were aware of its potential neurological consequences; similarly, 46.6% of those interviewed were aware of the possibility of hearing damage caused by certain medicines. Additionally, 60% of the respondents recognized that hearing loss can affect individuals of all ages, and 61.8% knew that audiometric tests evaluate hearing ability. However, 51.9% of the respondents expressed uncertainty about the threshold of risk to hearing and the limit values for noise pollution (49.2%).

Table 3 describes the attitudes of the respondents towards hearing health. The results show that 34.3% of the participants agreed that wearing earplugs is uncomfortable, while 35.5% disagreed. Moreover, 52.9% of the respondents believed that reading the package insert of medications is not useless, and 46.9% disagreed that it is best to dry hair in the sun. Furthermore, 34.4% of the respondents agreed that loud music gives them the right energy, while 39.4% disagreed. Also, 34.5% of the participants thought that earphones are more comfortable than speakers, while 35.3% did not.

Table 4 shows the frequencies of responses regarding behaviors associated with hearing health. Regarding ear hygiene, about 32.9% of the respondents reported using cotton buds for ear hygiene, while 50.4% reported not using ear sprays and 46.7% reported drying their ears with a clean towel after bathing. Moreover, 56.4% of the respondents reported never using earplugs, and 45.3% reported turning down the volume of the TV or radio during a conversation. Additionally, 56.6% of the respondents reported never falling asleep while listening to music with earphones. Although 80% of the respondents go to quiet places to rest from high sound levels during events, a significant percentage (68.1%) stand near speakers during a party. Interestingly, slightly more than half of the respondents (54.7%) had a visit related to hearing health in the last year.

Table 5 describes the results of the multiple logistic regression analysis (MLRA) in the three models. In Model I, an association was observed between knowledge regarding hearing health (used as an independent variable) and age, smoking habits, marital status, and education. Model II displays a statistically significant association between attitudes towards hearing health and non-smoking habits, education, and knowledge. In Model III, a statistically significant association was observed between behaviors and age, sex, having children, knowledge, and attitudes.

**Figure 1:**
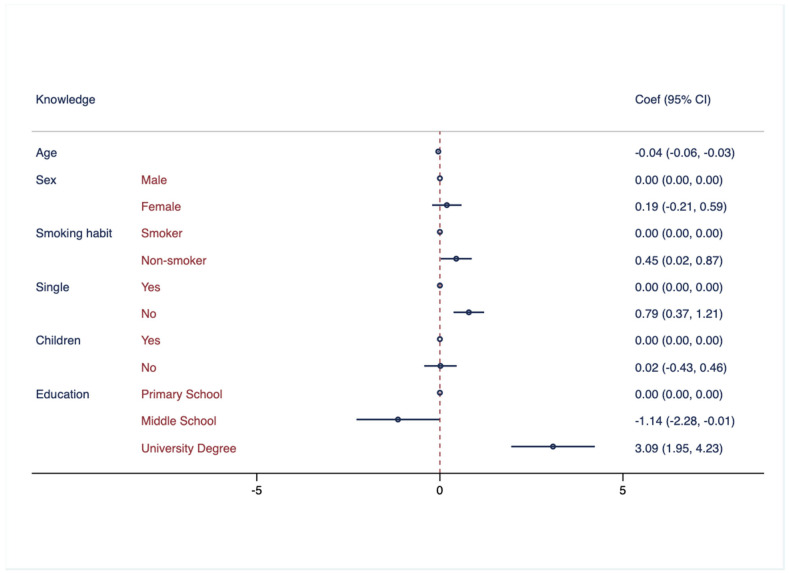
Association between knowledge regarding hearing health and demographic characteristics. Multivariate logistic regressions were employed, including knowledge regarding hearing health as outcome variable and controlled for the following variables: age, sex, smoking habits, marital status, having children, and education attainment. Results are presented as Coef. and CI.

Figure 1 presents an association analysis between knowledge of hearing health and various demographic factors, such as age, sex, smoking habits, marital status, having children, and education.

**Figure 2:**
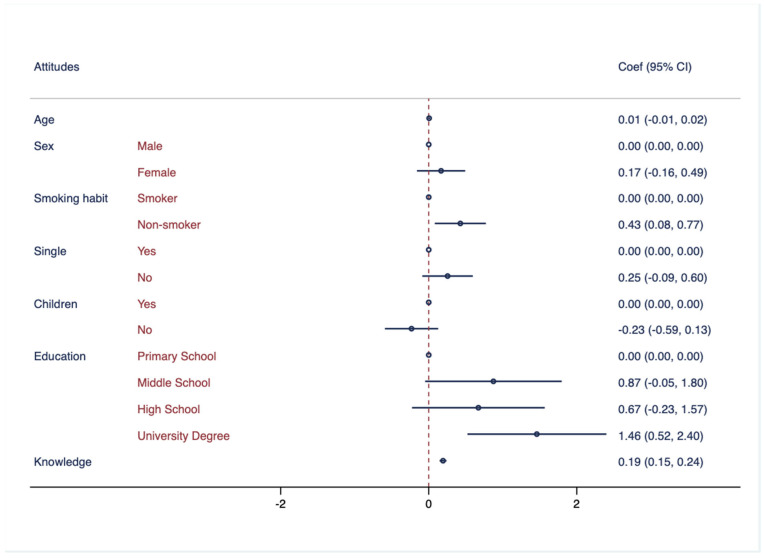
Association between attitude regarding hearing health and demographic characteristics. Multivariate logistic regressions were employed, including knowledge regarding hearing health as outcome variable and controlled for the following variables: age, sex, smoking habits, marital status, having children, education attainment, and knowledge. Results are presented as Coef. and CI.

Figure 2 illustrates the association between attitudes towards hearing health and several demographic factors, including age, sex, smoking habits, marital status, having children, and education attainment.

**Figure 3:**
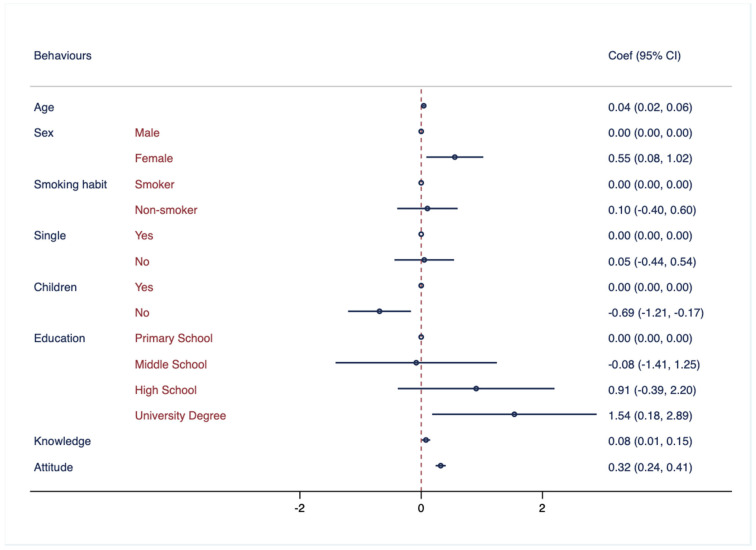
Association between behaviors regarding hearing health and demographic characteristics. Multivariate logistic regressions were employed, including knowledge regarding hearing health as outcome variable and controlled for the following variables: age, sex, smoking habits, marital status, having children, education attainment, knowledge, and attitude. Results are presented as Coef. and CI.

In Figure 3, the association between behaviors related to hearing health and several demographic factors, including age, sex, smoking habits, marital status, having children, and education, was examined. 

## 4. Discussion

The findings suggest a significant association between hearing health knowledge and young age, being single, and higher education attainment.

The first evidence suggests that younger people have better knowledge about hearing health. The findings are consistent with a study conducted by Khandekar [26] among people over 20 years old in the Omani population, which also found that young adults had a higher level of knowledge about hearing health. However, it is important to note that research findings may not always be consistent across different populations or settings. For example, a study conducted among iron and steel factory workers in Tanzania [27] showed no significant differences in the mean score of knowledge for age. This discrepancy could be due to various factors such as the specific demographics of the sample, occupational exposure to noise, cultural factors, or the accessibility of hearing health information and services in different regions.

Another result of this study was the association between being single and knowledge regarding hearing health. Although there is no previous research specifically examining this relationship, the previous literature indicates marital status as a proxy for attention to hearing health [28].

In addition, there is evidence of an association between education attainment and knowledge: college graduates have more knowledge about hearing health, which is consistent with a study by Sørensen [29], who found that individuals with higher levels of education were more likely to have their hearing tested, seek treatment for hearing loss when needed, and use hearing healthcare services. However, it is important to acknowledge that the relationship between educational attainment and hearing health knowledge may not be consistent across all populations or contexts. For instance, a study conducted among iron and steel factory workers in Tanzania [27] showed no significant differences in the mean score of knowledge for educational attainment. This discrepancy could be attributed to various factors, such as the specific educational system in Tanzania, the nature of the occupation, or the availability of hearing health education programs in the workplace. One potential explanation for the association between higher education levels and better knowledge about hearing health is that higher education is often associated with better health literacy overall [30,31], including knowledge about hearing health and the importance of seeking treatment when necessary. 

Therefore, promoting education and increasing awareness about hearing health, mainly among the elderly population, can have a positive impact on both education and health outcomes. Further research is needed to better understand the relationship between educational attainment and hearing health knowledge and develop effective interventions to promote hearing health among all individuals.

The second piece of evidence of the present study highlights an association between non-smoking habits and attitudes toward hearing health. However, there is a lack of research on the specific relationship between smoking habits and attitudes towards hearing health. This outcome is novel in the existing literature.

Furthermore, the study revealed a noteworthy association between knowledge and attitude, suggesting that knowledge regarding hearing health may contribute to the development of positive attitudes towards hearing health. This finding is consistent with previous research, including a study by Almutairi [22] that examined the general population of Saudi Arabia, as well as a study by Chung [32] that focused on adolescents and young adults. The interplay between attitudes and knowledge can significantly impact the diagnosis, intervention, and management of hearing loss. Therefore, it is crucial to further investigate the relationship between attitudes, knowledge, and hearing loss to devise effective strategies for addressing this concern.

The findings revealed a significant association between positive behaviors towards hearing health and older age, female gender, and good knowledge of hearing health.

The results suggest that the older participants and women demonstrated slightly better behaviors. Interestingly, a population-based cohort study [33] conducted between 2006 and 2010 in the United Kingdom with a sample size of over 500,000 individuals reported an association between female sex and better behaviors but not among older participants, who, however, may be aware of the problem and tend to recognize its severity, in contrast to the results of our study.

The study revealed an association between a higher level of knowledge about hearing health and the adoption of healthy behaviors. Furthermore, individuals who displayed positive attitudes towards hearing health were more inclined to adopt healthy practices. For instance, in a study conducted by Crandell [34], it was discovered that those who used hearing protective devices possessed better knowledge of hearing loss compared to those who did not use such devices. Similarly, Manchaiah [35] found that the knowledge and attitude of young adults towards music influenced their listening habits and risk of developing hearing loss, indicating that individuals who had better knowledge of hearing loss and a more favorable attitude towards hearing protection were more likely to engage in safe listening practices.

## 5. Limitations

The main limitation of this study was the reliance on self-reported behaviors through the use of questionnaires. This may have led to social desirability bias as respondents may have felt pressure to provide socially acceptable answers. However, we took steps to mitigate this bias by assuring the participants of anonymity and confidentiality. The questionnaire used in this research did not include specific sections to gather sociodemographic data, cultural information, type of families, socioeconomic levels, or studies of the parents. As a result, we were unable to provide in-depth insights into these aspects of the sample. While these factors may play a role in shaping the outcomes of the study, their absence limits the comprehensive understanding of the broader context surrounding our findings. Future studies could benefit from incorporating additional sections in the questionnaire to capture a more extensive range of participant characteristics and allow for a more comprehensive analysis. Also, being relatively new as topic in the literature, our discussions were limited to existing evidence.

## 6. Policies

The present study provides compelling evidence that both knowledge and attitude play important roles in promoting hearing health and hygiene practices. Specifically, individuals with greater knowledge about hearing health tend to exhibit more positive attitudes towards hearing protection and engage in better behaviors to protect their hearing. Thus, enhancing people’s knowledge of hearing health represents a potentially effective strategy to prevent hearing loss and related problems [36,37].

The results suggest that educational programs can be made more efficient by focusing on specific aspects of hearing health, particularly in older populations with smoking habits and lower educational attainment. Furthermore, interventions that address attitudes towards hearing health are likely to be even more effective in promoting good hearing hygiene practices. These interventions should be targeted towards high-risk populations such as men, smokers, and individuals with low educational levels who exhibit worse attitudes towards hearing health.

Improving knowledge and attitudes towards hearing health can promote better hearing hygiene practices and prevent hearing loss, especially in vulnerable populations. Therefore, public health policies that aim to promote hearing health and prevent hearing-related disabilities should prioritize the development and implementation of evidence-based educational interventions tailored to the specific needs of different population groups. By addressing knowledge gaps and promoting positive attitudes towards hearing health, these interventions could help improve hearing-related behaviors and prevent hearing loss in the long run.

## 7. Conclusions

Raising awareness about hearing health is an urgent and critical need that demands immediate attention. The study has shed light on important factors associated with better knowledge, attitudes, and behaviors towards hearing health. Younger age, being single, and higher education attainment were found to be significantly correlated with improved knowledge about hearing health. Similarly, positive attitudes towards hearing health were associated with non-smoking habits and better knowledge on the subject. Additionally, positive behaviors towards hearing health were correlated with older age, female gender, and a good understanding of hearing health.

These findings, aligning with the study’s aim, hold substantial significance for assessing hearing behaviors and carry important implications for public health policies. The data gathered can be instrumental in designing targeted interventions to enhance hearing health and address issues related to hearing loss. By implementing such policies, we can effectively improve overall hearing health and well-being for individuals and communities alike. It is evident that addressing hearing health should be prioritized in public health agendas to prevent and manage hearing-related challenges effectively.

## Figures and Tables

**Table 1:** Study population demographic characteristics.

Study Population	N	Percentage
**Sex**	(1155)	
Male	540	46.7
Female	615	53.3
**Age**		
<30	455	39.4
31–35	123	10.6
36–40	84	7.3
41–45	80	6.9
46–50	61	6.9
>51	333	28.8
**Education**		
Primary school	48	4.2
Middle school	172	14.9
High school	501	43.4
University degree	434	37.6
**Children**		
Yes	457	39.6
No	698	60.4
**Smoking habits**		
Yes	413	35.8
No	742	64.2
**Marital Status**		
Single	468	40.5
In a relationship	687	59.5

**Table 2:** Knowledge of respondents regarding hearing health.

N.	Statement (Variables)	Agree (%)	Uncertain (%)	Disagree (%)
K1	The ear is the organ of hearing.	68.8	14.4	16.8
K2	Cotton swabs are the safest way to clean your ears.	17.4	26	56.6
K3	The removal of the earwax plug must be performed by the doctor.	59.3	22.9	17.7
K4	Scuba diving is dangerous for hearing.	51.3	30.0	18.6
K5	Some medicines can cause hearing damage.	46.6	39	14.4
K6	Otitis media is an ear disease.	67.4	17.6	15
K7	Otitis media can have neurological consequences.	46.9	38.4	14.6
K8	Hearing loss only affects older people.	15.8	24.2	60
K9	The audiometric test evaluates hearing ability.	61.8	19.3	18.9
K10	The unit of sound measurement is the decibel (dB).	62.3	22.7	15
K11	The risk threshold for hearing is about 80dB.	32.9	51.9	15.2
K12	The limit values for noise pollution are defined by the DPCM * of 14 November 1997.	33.7	49.2	17.1

* Decree of the Prime Minister (DPCM).

**Table 3:** Attitude of respondents toward hearing health.

N.	Statement (Variables)	Agree (%)	Uncertain (%)	Disagree (%)
A1	It is uncomfortable to wear earplugs.	34.4	30.1	35.5
A2	It is important to go for a morning run.	56.3	25.4	18.3
A3	It is nice to go clubbing with friends.	41.7	26.6	31.7
A4	Loud music gives the right energy.	34.4	26.2	39.4
A5	It is nice to have the TV on during meals.	44.4	25.1	30.5
A6	Earphones are more comfortable than speakers.	34.5	30.2	35.3
A7	In summer it is best to dry your hair in the sun.	29.3	23.7	46.9
A8	It is important to always have a sanitizer with you.	62.2	20.3	17.5
A9	Reading the package insert of the medicines is useless.	27.5	19.6	52.9
A10	It is useful to have your teeth cleaned every six months.	58.1	22.4	19

**Table 4:** Behaviors of respondents concerning hearing health.

N.	Questions	Yes/Always (%)	Often (%)	Sometime (%)	Never (%)
B1	Do you use cotton buds to clean your ears?	32.90	16.36	26.67	24.07
B2	Do you use ear sprays to clean your ears?	16.71	11.95	20.95	50.39
B3	Dry your ears with a clean towel after the bath?	46.67	16.71	17.32	19.31
B4	Do you swim in the pool or in the sea even if it is cold?	20.17	10.82	26.41	42.60
B5	Do you use earplugs?	11.00	15.32	17.32	56.36
B6	During a conversation, turn the volume down TV or radio?	45.28	23.90	17.66	13.16
B7	Do you happen to see fireworks shows up close?	20.35	16.10	40.52	23.03
B8	During events, do you go to quiet places to rest from high sound levels?	24.24	21.13	34.72	19.91
B9	Do you happen to stand near the speakers during a party?	18.87	12.21	36.97	31.95
B10	Do you turn up the audio volume with earphones?	24.07	24.07	28.23	23.64
B11	Do you fall asleep listening to music with earphones?	11.26	10.04	22.08	56.62
B12	In the last year, have you had a hearing check-up?	20.09	10.91	14.29	54.72

**Table 5:** Results of the linear multiple regression analysis (MLRA).

	Coefficients Not Standardized		Coefficients Standardized			
	b	Standard Error	t	95% Conf. Interval		*p*-Value
**Model I—Dependent Variable: Knowledge**						
*Prob > F = 0.000*	*R-squared = 0.4744*			*Root-MSE = 3.4311*		
Age	−0.044	0.007	−5.47	−0.059	−0.027	0.000
Sex	0.190	0.204	0.93	−0.211	0.592	0.352
Single	0.790	0.213	3.71	0.372	1.21	0.000
Children	0.016	0.226	0.07	−0.427	0.459	0.943
Smoking habits	0.446	0.216	2.06	0.022	0.871	0.039
Education *						
Middle School	−1.14	0.578	−1.98	−2.28	−0.009	0.048
High School	−3.25	0.554	−5.87	−4.34	−2.16	0.000
University Degree	3.09	0.582	5.31	1.95	4.23	0.000
**Model II—Dependent variable: Attitudes**						
*Prob > F = 0.000*	*R-squared = 0.1710*			*Root-MSE = 2.7859*		
Age	0.005	0.007	0.80	−0.008	0.018	0.424
Sex	0.166	0.166	1.00	−0.160	−0.493	0.032
Single	0.254	0.174	1.46	−0.867	0.596	0.144
Children	−0.232	0.263	−1.27	−0.592	0.127	0.205
Smoking habits	0.426	0.175	2.43	0.081	0.772	0.015
Education *						
Middle School	0.874	0.470	1.86	−0.049	1.80	0.063
High School	0.671	0.457	1.47	−0.226	1.57	0.142
University Degree	1.46	0.478	3.05	0.523	2.40	0.002
Knowledge	0.193	0.024	8.05	0.145	0.240	0.000
**Model III—Dependent variable: Behavior**						
*Prob > F = 0.000*	*R-squared = 0.1201*			*Root-MSE = 4.0105*		
Age	0.042	0.009	4.44	0.023	0.060	0.000
Sex	0.554	0.239	2.31	0.084	1.02	0.021
Single	0.050	0.251	0.20	−0.441	−0.542	0.841
Children	−0.687	0.264	−2.60	−1.20	−0.169	0.009
Smoking habits	0.103	0.254	0.41	−0.395	0.601	0.685
Education *						
Middle School	−0.082	0.678	−0.12	−1.41	1.25	0.904
High School	0.906	0.658	1.38	−0.385	2.20	0.169
University Degree	1.54	0.691	2.22	0.180	2.89	0.026
Knowledge	0.079	0.035	2.22	0.009	0.148	0.027
Attitude	0.322	0.042	7.56	0.238	0.405	0.000

* Primary School as Reference.

## Data Availability

Not applicable.

## Supplementary Materials

The following supporting information can be downloaded at: https://www.mdpi.com/article/10.3390/healthcare11162253/s1, The questionnaire used in this study.
