# Supplementary material for: Understanding Hearing Health: A Cross-Sectional Study of Determinants in a Metropolitan Area
Source: Healthcare (Basel). 2023 Aug 10;11(16):2253. doi: 10.3390/healthcare11162253 (PMC10454096; doi:10.3390/healthcare11162253)
Supplement: Supplementary file 1 [file healthcare-11-02253-s001.zip › healthcare-2485783-supplementary.pdf]

The questionnaire used in this study.

Anonymous questionnaire on knowledge, attitudes, and behaviors regarding hearing health.

Participant n°: ____________ Date: ___________________

(Please write X in front of the proper answer)

Age Gender: □ M □ F

Do you smoke? □ Yes □ No Are you single? □ Yes □ No

Do you have children? □ Yes □ No If yes, how many?__________

Educational level:

□ Primary School □ High School

□ Middle School □ University Degree

Knowledge	Agree	Uncertain	Disagree
**1.** The ear is the organ of hearing.			
**2.** Cotton swabs are the safest way to clean your ears.			
**3.** The removal of the earwax plug must be performed by the doctor.			
**4.** Scuba diving is dangerous for hearing.			
**5.** Some medicines can cause hearing damage.			
**6.** Otitis media is an ear disease.			
**7.** Otitis media can have neurological consequences.			
**8.** Hearing loss only affects older people.			
**9.** The audiometric test evaluates hearing ability.			
**10.** The unit of sound measurement is the decibel (dB).			
**11.** The risk threshold for hearing is about 80dB.			
**12.** The limit values for noise pollution are defined by the DPCM * of 14 November 1997.			

Attitudes	Agree	Uncertain	Disagree
**1.** It is uncomfortable to wear earplugs.			
**2.** It is important to go for a morning run.			
**3.** It is nice to go clubbing with friends.			
**4.** Loud music gives the right energy.			
**5.** It is nice to have the TV on during meals.			
**6.** Earphones are more comfortable than speakers.			
**7.** In summer it is best to dry your hair in the sun.			
**8.** It is important to always have a sanitizer with you.			
**9.** Reading the package insert of the medicines is useless.			
**10.** It is useful to have your teeth cleaned every six months.			

BEHAVIORS	YES	OFTEN	SOMETIME	NEVER
**1.** Do you use cotton buds to clean your ears?				

**2.** Do you use ear sprays to clean your ears?				
**3.** Dry your ears with a clean towel after the bath?				
**4.** Do you swim in the pool or in the sea even if it is cold?				
**5.** Do you use earplugs?				
**6.** During a conversation, do you turn the volume down on the TV or radio?				
**7.** Do you happen to see fireworks shows up close?				
**8.** During events, do you go to quiet places to rest from high sound levels?				
**9.** Do you happen to stand near the speakers during a party?				
**10.** Do you turn up the audio volume with earphones?				
**11.** Do you fall asleep listening to music with earphones?				
**12.** In the last year, have you had a hearing check-up?				
